# Olfactory bulb network dynamics as a pattern reservoir for adaptive cortical representations

**DOI:** 10.1186/1471-2202-14-S1-P422

**Published:** 2013-07-08

**Authors:** Maximilian Puelma Touzel, Michael Monteforte, Fred Wolf

**Affiliations:** 1 Max Planck Institute for Dynamics and Self-Organization, Goettingen, Germany; 2 Faculty of Physics, Georg-August-University, Goettingen, Germany; 3 Bernstein Center for Computational Neuroscience, Goettingen, Germany

## 

Dynamic instabilities have been offered as an explanation for how the olfactory bulb can separate its inputs[1]. However, models that can flexibly adjust both to separate and categorize inputs based on relevance are lacking. Dynamic stability of networks with linear coupling is determined exclusively by the Lyapunov spectrum, while nonlinear 'spike' coupling adds an instability with respect to changes in spike sequence. When the Lyapunov spectrum is stable (e.g. for rapid action potential onset), the resulting stable chaos is determined by a critical scale separating coexisting stable and unstable dynamics [2]. Here, we explore how this feature can be used by a network modeled after the bulb to facilitate learning of inputs.

We built a spiking network model of the predominately inhibitory mitral-granule cell network. Using our analytic solution to a 2D mitral cell model, we implemented an efficient and precise root finding algorithm to obtain the next spike time within a network. Using this, we performed numerically exact, event-based network simulations, iterating from one spike in the network to the next. By varying a parameter that controls the intrinsic frequency, we explore the network dynamics from integrator to resonator units, ending with a network that replicates the zebrafish bulb's first and second order spiking statistics and the network oscillation it exhibits upon being driven by sensory input. To study the effects of the intrinsic resonance on the resulting network oscillation, we analytically derived the firing rate response of the single neuron to weak oscillatory synaptic input and show a resonance that depends on the intrinsic frequency. Then, to assess the stability of such networks, we analytically calculated the single spike Jacobian of the event map, which describes how small perturbations evolve between spikes and which we use to compute the full Lyapunov spectrum, finding it stable. An analysis of stability with respect to larger size perturbations, in particular to cases of temporally varying input rates, shows that periods of activity are much more sensitive and that the oscillation can modulate that sensitivity. This finding suggests a scheme for controlling the operating point of the system along the trade-off between categorizing and separating inputs to facilitate learning by olfactory cortex[3]. So, finally we explore such a scheme by having downstream neurons learn families of trajectories from a given input.
